# Alcohol Consumption Modifies Susceptibility to HIV-1 Entry in Cervical Mucosa-Derived CD4+ T cells of Women Resident in a Fishing Community of Lake Victoria, Uganda

**DOI:** 10.21203/rs.3.rs-3210670/v1

**Published:** 2023-08-24

**Authors:** Bagaya Bernard Ssentalo, Emmanuella Driciru, Muwanda Fahad, Mary Nantongo, Marion Namuleme, Paul Kato Kitandwe, Enoch Muyanja Ssekayita, Ronald Galiwango, Violet Mirembe, Barbarah Kawoozo Muwenda, Moses Muwanga, Alex Kayongo, Fredrick Lutwama

**Affiliations:** Makerere University; Makerere University; Makerere University; Makerere University; UVRI-IAVI-HIV Vaccine Program; UVRI-IAVI-HIV Vaccine Program; UVRI-IAVI-HIV Vaccine Program; Rakai Health Sciences Program; UVRI-IAVI-HIV Vaccine Program; UVRI-IAVI-HIV Vaccine Program; Entebbe General Referral Hospital; Makerere University; Makerere University

**Keywords:** HIV-1, Susceptibility, CD4+ T cells, Cervical mucosa, Alcohol Consumption

## Abstract

**Background:**

A significant overlap exists in the burden of Alcohol Use Disorders (AUDs) and the HIV epidemic in Sub-Saharan Africa. Over 60% of HIV infections occur in women, mostly through the cervical mucosa. Absorption and systemic circulation of alcohol induces global physiological and immune effects, including at the genital mucosa. Alcohol alters expression of cell surface receptors, mucosal barrier permeability, inflammatory responses, and lymphocyte trafficking and homing. However, a substantial knowledge gap exists on whether these cellular and or immunological effects of alcohol modify the consumers’ CD4+ T cell susceptibility to HIV-1 entry at the cervical mucosa. HIV seronegative women, aged 18–49 years were recruited from Kasenyi and Kigungu fish landing sites of Lake Victoria. They were categorized as Alcohol Consumers (n=27) or non-Alcohol Consumers (n=26) based on the World Health Organization Alcohol-Use-Disorder-Test (WHO-AUDIT) at a cut-off score of >=8/40 and <8/40, respectively. Cytobrush-collected Cervical Mononuclear Cells [CMCs] and Peripheral Blood Mononuclear Cells [PBMCs] from heparinized whole-blood were surface stained for CD4+ T cell immunophenotyping. To measure susceptibility to HIV entry, CMCs and PBMCs were co-cultured overnight with equal amount of GFP-tagged HIV-1 pseudo-virus particles. Both immunophenotyping and HIV entry were measured on a BD LSR II flow cytometer.

**Results:**

There was no significant difference in the frequency of CD4+ T cells in blood (p=0.451) or mucosa (p=0.838) compartments across study groups. However, we observed a combined four-fold higher HIV entry (p=0.0001) into cervical versus blood-derived CD4+ T cells regardless of alcohol consumption status. More so, cervical-derived CD4+ T cells of alcohol-consumers showed a two-fold increase in susceptibility to HIV entry (P=0.0185) compared to the non-alcohol consumer group. Double positive α4β7+CD4+T cells of alcohol consumers exhibited a higher HIV entry compared to those from alcohol non-consumers(p=0.0069).

**Conclusion:**

This study demonstrates that cervical CD4+ T cells are more susceptible to HIV entry than those from blood. Also, cervical CD4+ T cells of alcohol consumers are more susceptible than those of non-consumers. Differences in frequencies of α4β7+ CD4+ T between alcohol consumers and non-consumers’ cells may account for the increased susceptibility to HIV entry.

## Background

Despite reductions in new HIV infections in the last decade, Sub-Saharan Africa still bears a disproportionately high burden of HIV/AIDs; accounting for over 70% of the 37.9 million global HIV infections [1]. The high HIV transmission rates within the “most-at-risk-populations” (MARPs) significantly contributes to the high HIV/AIDS burden [2]. MARPs act as reservoirs of HIV/AIDS that sustain seeding of the virus into the general population. Therefore, tackling HIV transmission among MARPs is fundamental to achieving the WHO’s 90-90-90 goals [3]. The most notable of these MARPs in Sub-Saharan Africa are the commercial sex workers, migrant workers, long-distance truckers and the dwellers of fishing communities (Fisherfolk) [4].

HIV prevalence in fishing communities is 4–14 fold higher than that in the general population [5–7], justifying the refocussing of prevention interventions to accelerate reductions in incidence. However, factors such as poor access to health services, poor or absolute lack of roads and or reliable water transport, low levels of education and the island nature of most fishing villages disadvantages these communities, making access to these HIV/AIDS interventions dismal to-date. In fact an estimated 22–40% of fisherfolk in Uganda are thought to be HIV infected significantly contributing to the the national HIV prevalence rate that stands at 5.7% [8]. Although many socio-behavioural factors have been associated with this disproportionate rate of HIV/AIDS among the fisherfolk, alcohol consumption is by far the most outstanding, with up to 64% of all incident HIV among the fisherfolk of Lake Victoria recently being attributed to it [9]. Uganda ranks among the top alcohol consumer per capita countries in the world, with a national alcohol use disorders prevalence of 52% [10]. Among Uganda’s fisherfolk population however, the prevalence of hazardous alcohol consumption is as high as 53–70% [9]. Hence, HIV prevention interventions that integrate addressing of alcohol use disorders among the fisherfolk will be required to maximize impact.

Socio-behavioural and epidemiological studies on role of alcohol consumption on HIV transmission have largely concluded on alcohol’s alteration of judgment and decision-making, reduction of one’s perception of risk. In addition, venues where people drink may also be places where casual or transactional sex can easily be obtained, and those who are intoxicated may forget about or be unable to use condoms [11–13]. However, in our opinion alcohol and HIV interplay surpasses these socio-behavioural factors and has a significant biological component that must be understood to facilitate the design of integrated prevention interventions. Alcohol readily absorbs into blood, immediately inducing systemic physiological and immune modulatory effects, including at the genital mucosa where most heterosexual exposures occur [14, 15]. Documented alcohol’s biological effects among others include impairment of T cell function, acceleration of HIV replication in PBMCs [16] and alteration of expression of cell surface receptors and cytokines[17]. Skewing of T-cell phenotype distribution towards memory T-cells and the decrease of T-cell population frequencies due to alcohol-induced T-cell apoptosis have also been documented [18]. Importantly alcohol is thought to induce inflammatory responses at mucosal surfaces, affecting integrity of tight junctions in the gut and other mucosal barriers [19]. Mucosal barrier disintegration exacerbates microbial translocation across membranes and into blood circulation, resulting in a state of chronic inflammation [20]. Additionally, these chronic inflammatory responses may trigger trafficking and homing of HIV-susceptible CD4 + T cells to local mucosal environment, most likely increasing the risk of HIV infection upon exposure [19, 21]. Nonetheless, a substantial knowledge gap still exists to directly support the concept that alcohol mediates modification of susceptibility to initial HIV-1 entry at the genital mucosa and the underlying mechanisms that lead to the establishment of a productive infection. Unfortunately, a high fraction (≈ 60%) of the new HIV infections globally occur in women as a result of social, behavioural, cultural, structural and biological factors that pre-dispose and or enhance their susceptibility to HIV acquisition [9]. More so, women in the fishing communities live in a subordinate economic and social position, coupled with high risk sexual behaviours and social environment that precipitates their vulnerability to HIV infection [22]. In addition, women tend to have a lower peak-alcohol-level in systemic circulation compared to men [23], resulting in more severe socio-behavioural and biological consequences of alcohol use [23, 24]. Therefore, we set out to purposively choose a female study population among fishing communities of Lake Victoria, Entebbe peninsula, in Uganda, to investigate the effects of alcohol consumption on modification of susceptibility to HIV-1 entry in cervical mucosa-derived CD4 + T cells. The findings of this study are crucial to inform the development and or modification of existing HIV/AIDS prevention public health strategies that target HIV transmission at the genital mucosa in women.

## Results

The baseline characteristics between alcohol- and non-alcohol consumer study groups were comparable. The Mean age in years in both study groups was 29 years with 90% of the participants reporting vaginal sex in the last 3 months (Table 1). Majority of the participants (88.5% of the alcohol consumers and 84.0% of the non-alcohol consumers) did not exhibit symptomatic genital tract infections as shown in Table 1.

**Figure 1:**
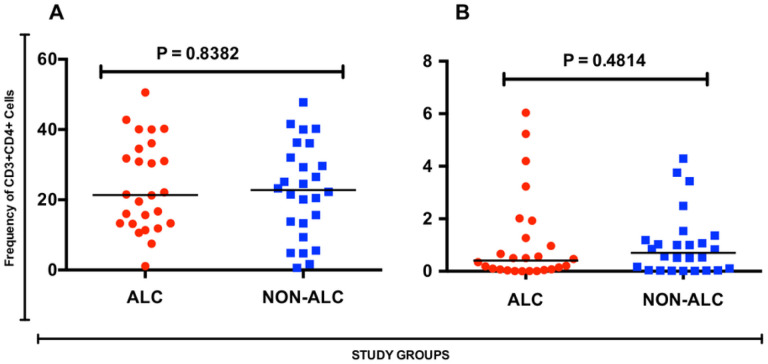
Frequency of CD3+CD4+ T helper cells among alcohol consumers and their non-alcohol consumer counterparts. [A] Frequency in the Blood compartment, [B] Frequency in the cervical compartment

Since HIV-1 is known to infect CD4 + T cells, and alcohol use among other biological effects may affect CD4 + T cell numbers, any significant differences in either of the blood or cervical compartments might lead to differences in HIV susceptibility between alcohol consumer and non-consumer counterparts. However, we found that phenotypic frequency of CD4 + T cell population was comparable between the alcohol consumers and non- consumer groups, in both the cervical mucosa (Fig. 1A, p = 0.838) and the blood compartment (Fig. 1B, p = 0.451).

**Figure 2:**
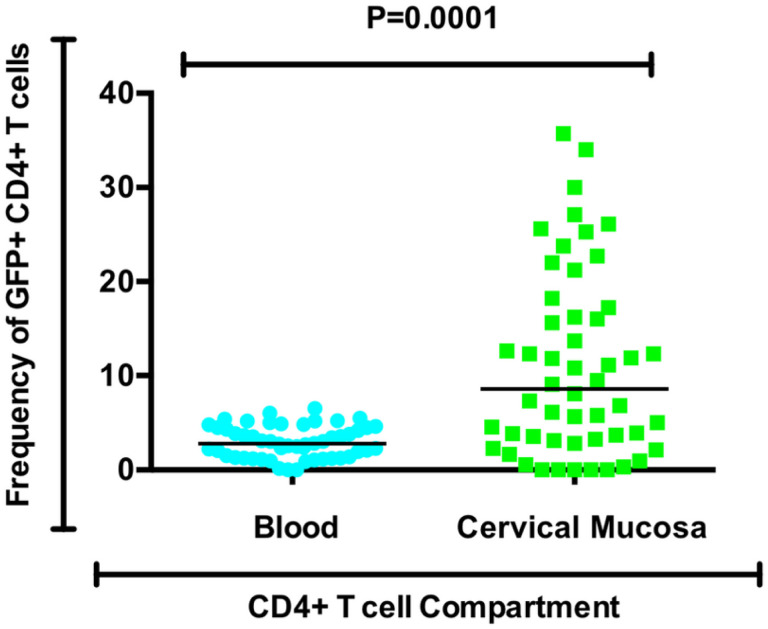
Frequency of GFP+CD4+T cells from the blood compartment compared to cervical mucosa compartment regardless of alcohol Use behaviour.

We went on to determine whether mucosa-derived and blood CD4 + T cells were comparable in their permissiveness to pseudovirus entry. We found that regardless of alcohol consumption status of the participants, HIV pseudovirus entry into cervical CD4 + T cells was up to four times higher (p = 0.0001) than entry into blood-derived CD4 + T cells (Fig. 2).

**Figure 3:**
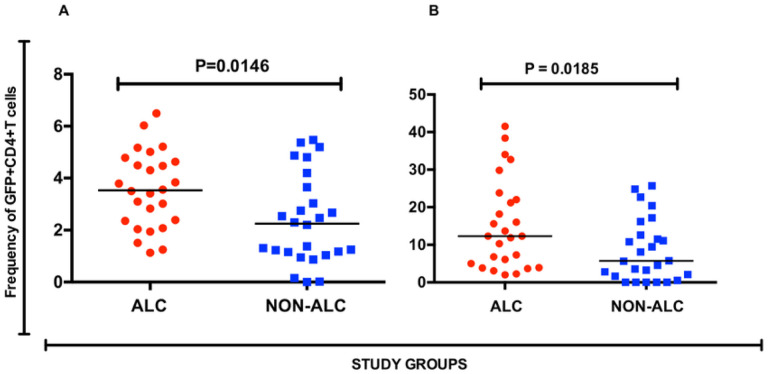
Frequency of GFP+ CD4+ T helper cells among alcohol consumers and their non-alcohol consumer counterparts. [A] Frequency in the Blood compartment, [B] Frequency in the cervical compartment

HIV-1 transmission in Uganda is predominantly transmitted heterosexually via genital mucosa. Differences in permissiveness to HIV entry at the cervical mucosa CD4 + T cells could help explain increased HIV-1 rates among alcohol consumers. When we compared HIV-pseudovirus entry into CD4 + T cells from the cervical compartment of alcohol consumer and their non-consumer counterparts, we found a that entry was significantly higher (P = 0.0185) in the former group (Fig. 3).

**Figure 4:**
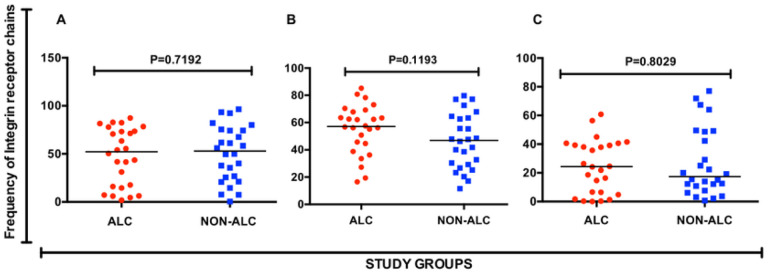
Frequency of integrin receptor chain expression by T helper cells in the cervical mucosa. [A] Frequency of α4+, [B] β7+ and [C] β1+ T helper cells

**Figure 5:**
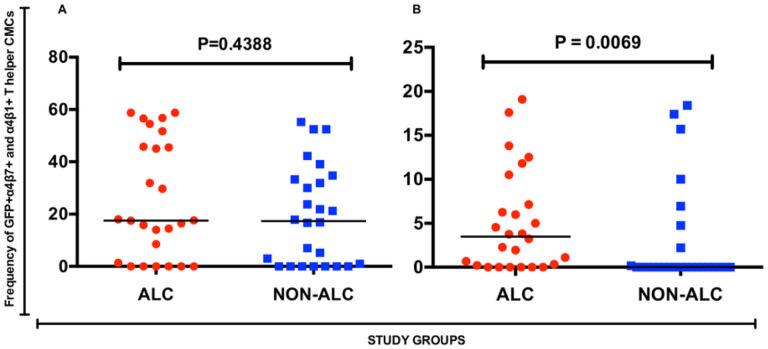
Frequency of GFP+ integrin receptor expressing cervical mucosa-derived T helper cells. [A] Frequency of GFP+α4β7+ T helper cells, and [B] Frequency of GFP+ α4β1+ T helper cells

We did not observe a significant difference in the frequencies of α4 (p = 0.719), β1 (p = 0.803) and β7 (p = 0.119) CMC-derived CD4 + T cells of alcohol consumers and non-alcohol consumer groups (Fig. 4). We also found no significant difference (p = 0.4388) in HIV entry (frequency of GFP expression) in double-positive α4β1 + CD4 + T cells between the two study groups (Fig. 5B). However, double positive α4β7 + CD4 + T cells from the alcohol consumer group exhibited a significantly increased (p = 0.0069) HIV entry in the alcohol consumer group than in the non- consumer group (Fig. 5A), despite the comparable frequency in cell phenotype.

## Discussion

In this study, we investigated the impact of alcohol consumption on CD4 + T cell *In Vitro* susceptibility to HIV-1 pseudovirus entry. HIV pseudovirus entry was 2-fold higher in cervical CD4 + T cells obtained from alcohol consumers compared to those from alcohol non-consumers. Generally, CD4 + T cells from the cervical mucosa exhibited a higher susceptibility (four-fold) to HIV entry compared to those from the blood compartment regardless of alcohol consumption status. In a similar study, Joag et al, reported a 2.8-fold increase in HIV entry into cervical T cells compared to PBMC derived CD4 + T cells. A further 7.7 fold increased entry into CMCs was reported when CMC and PBMCs were matched for actual CD4 + T cell input [25]. Mucosal CD4 + T cells are typically highly activated cell types, being enriched in CD38 and CD69 acute markers for T cell activation and low in memory marker CD27 expression compared to the blood compartment. This activated state enhances HIV entry given that the virus usually exhibits a preferential infection of the activated cells [26]. This explains the observed higher susceptibility of the CD4 T cells from the cervical mucosal compartment to HIV entry compared to the blood compartment. On the other hand, the high HIV entry into cervical T cells could also be due to the role of HIV-pulsed Dendritic cells in enhancing HIV infection in mucosal environments and in In Vitro co-cultures. It is to be noted that, in this study, cell sorting was not performed prior to In Vitro infection of cells with pseudo-virus. In the genital mucosa, Intraepithelial DCs are highly populated and are known to account for 90% of the HIV dissemination in cervical explants [27]. DCs bind, capture and traffic the HIV virus without themselves being infected and then form a virological infectious synapse with mucosal CD4 + T cells to transfer the virus to mucosal CD4 + T cells. DCs can also mediate HIV infection through *trans*-infection using DC derived exosomes as well as infection in *cis* [27]. However, the role of mucosal Dendritic cells in enhancing HIV entry was not investigated in this study.

Although to our knowledge, studies on the immunological impact of alcohol on the susceptibility of cervical T cells to HIV-1 infection have not been done before in Uganda, a previous study on blood derived cells reported significantly higher HIV-entry into PBMCs isolated from healthy individuals after consuming 0.7–3.1 litres of alcoholic beverages compared to entry before alcohol ingestion [16]. Given that we observed a comparable cervical CD4 + T cell frequency between alcohol and alcohol non-consumers suggests that alcohol induced mucosal cell trafficking may not account for the observed increase in HIV psudovirus entry in alcohol consumers. However, the increased biological susceptibility of alcohol consumers to infections, including HIV-1, has been attributed to the immunosuppressive effect of alcohol such as reduced lymphocyte numbers and dysfunction, and altered surface receptor gene expressions [21]. Further, alcohol up-regulates expression of receptors such as CCR5 and possibly Integrin receptors which are involved in the binding and capture of HIV virions to initiate cellular entry (infection) events [25]. PBMCs from alcohol consumers have also been shown to exhibit cellular dysfunctions as a result of impaired IL-2 production which plays a critical role in intercellular signalling [21]. These factors co-jointly play a significant role in promoting viral entry and subsequently increasing susceptibility of alcohol consumers to HIV infection. This, in addition to the social behavioural impacts of alcohol consumption that increase their risk of HIV acquisition.

In this study, we found that α4β1 integrin receptor expression results in increased HIV-entry among alcohol consumers. This finding agrees with a recent study that reported increased HIV infection in gut CD4 + T cells that exhibited high α4β1 expression [21]. Noteworthy, Emerging evidence indicates that α4β1 integrin receptor facilitates viral capture and binding on mucosal surfaces thus enhancing HIV entry into CD4 + T cells [28]. Furthermore, α4β1 expressing T cells have been found to exist in an activated state being rich in activation markers CD38 and CD69 and low in CD27 expression in addition to high CCR5 expression which favours HIV entry [25, 26, 28].

Contrary to our expectation, we did not observe any difference in the CD4 + T cell phenotype between the two study groups. Alcohol is known to result in alterations in T cell populations through mechanisms such as induction of T cell apoptosis and alteration of receptor gene expression through HSF-1 transcriptional factor [29–31]. However, several studies have also reported no significant differences in T cell phenotypes as observed in this study [16, 32]. Profound T cell decrease is usually attributed to chronic alcohol consumption and specific alcoholic beverages with high alcohol content such as Gins and “spirits”, attributes uncommon in female consumers [32].

A limitation of this study is that it is not possible to absolutely exclude the presence of blood derived CD4 + T cells from cervical CD4 + T cell populations. In addition, we did not include all markers of susceptibility in this study. However, despite these limitations, this is the first study to elucidate the immunological impact of alcohol ingestion on susceptibility to initial HIV-1 Infection at the cervical mucosa to our knowledge. Our findings do highlight and support the need for robust integration of alcohol-use control strategies and advocacy for alcohol abstinence into the existing HIV prevention programs in order to fortify efforts to reduce the HIV burden in Sub-Saharan Africa.

## Conclusions

this study demonstrates that cervical CD4 + T cells are more susceptible to HIV entry than those from blood. Also, cervical CD4 + T cells of alcohol consumers are more susceptible than those of non-alcohol consumers, and differences in frequencies of α4β7 + CD4 + T cells appeared to account for the increased susceptibility.

## Methods

### Study Design and Setting;

We conducted a cross-sectional study among women in the fishing communities of Kigungu and Kasenyi fish landing sites along the shores of Lake Victoria, Entebbe peninsula. Sample collection procedures were performed by a trained nurse at the Post Abortive Care Unit of Entebbe General Hospital. Samples were in under 3 hours, transported to and analysed at the UVRI-IAVI HIV Vaccine Program Laboratory located within the Uganda Virus Research Institute (UVRI), Entebbe.

### Study Population and Sampling;

Participants were females aged 18 to 49 years, HIV seronegative, not pregnant and must have been residents of the study area for at least 3 months prior to enrolling into the study. All participants gave a written informed consent before enrolment into the study. Eligible participants who were experiencing menstrual bleeding at their clinic visit were rescheduled approximately 7 days from the last day of menstrual bleeding. Female participants with an Intra-Uterine Device (IUD) were excluded due to a risk of the IUD dislodgement during the cytobrush sample collection procedure. Cytobrush samples with visible blood stains were excluded to avoid PBMC contamination of CMCs.

### Alcohol Use Disorder Identification Test (AUDIT) questionnaire administration and categorization of participants;

The standard WHO-AUDIT questionnaire was administered to all participants. Total AUDIT scores were calculated and participants were assigned as Alcohol Consumers (27 participants) or alcohol non-consumers (26 participants) based on WHO’s cut-off score of > = 8/40 and < 8/40 and respectively prior to sample collection.

### HIV Screening and Pregnancy testing;

HIV was tested using the Uganda Ministry of Health’s Rapid Diagnostic Test (RDT) algorithm. Briefly, samples were first tested on the Determine HIV-1/2 Ag/Ab Combo-Antibody test [6], non-reactive results were immediately reported [33]. Samples reactive on the Determine HIV-1/2 Ag/Ab Combo-Antibody RDT were tested on the HIV1/2 STAT-PAK RDT [7] and if reactive, results were reported as such. Samples with discordant results between Determine and STAT-PAK methods were tested on the SD Bioline HIV-1/2 Antibody test [1], the results of which were reported as final. Testing for pregnancy was done using the QuickVue One Step HCG Combo test (Quidel, CA) on the urine samples.

### Cervical Mononuclear Cells (CMCs) and Blood sample collection;

CMCs were collected using the Cytobrush (Fisher Scientific, USA). Two cervical Cytobrush samples (one for the phenotyping assay and another for the HIV-1 pseudo-virus infectivity assay) were collected from each consenting participant by a trained Nurse using a sterile Cytobrush under speculum examination. A Cytobrush was gently inserted into the cervical os and rotated at 360 degrees. The cytobrush was then immediately retracted and placed into 50ml of RPMI-1640 supplemented with 1%Penicillin/Streptomycin/Amphotericin B and 2% Fetal Bovine Serum (FBS) and placed on ice. Samples were transported to the laboratory on ice for processing within 3 hours of collection. Additionally, 10mls of heparinized venous blood sample was collected from each participant to serve as a comparator for CMCs.

### CMC processing;

CMC isolation was performed as described elsewhere [34]. In brief cytobrush samples were vortexed for 2 minutes. The cytobrush was then squeezed between the thumb and index finger, and subjected to several rotating motions while the tip was still in the media. The procedure was repeated in R10 media (RPMI 1640 supplemented with 10% FBS) for all the CMCs to be released into suspension. The CMCs in suspension were filtered through a 100μm nylon cell strainer (Fisher Scientific, USA) into a newly labelled 50 ml conical tube (BD, USA). This CMC suspension was centrifuged for 10 minutes at 500g without brakes. The supernant was gently disposed of and the CMC pellet resuspended by gentle agitation in PBS supplemented with 2%FBS. The Cytobrush sample of one participant (from the alcohol consumer group) was excluded from laboratory analysis due to visible blood contamination of the cytobrush sample.

### Peripheral Blood Mononuclear Cells (PBMC) separation;

PBMCs were isolated from heparinized blood using the Ficoll-Histopaque density gradient centrifugation method [35]. Briefly, 10mls of anti-coagulated blood was gently layered over 10mls of Ficoll-Histopaque solution in a 50ml conical tube (BD, USA) and centrifuged at 400xg, 22^0^C for 40 minutes. The mononuclear cell layer that is between the plasma layer and Ficoll-Histopaque layer was carefully pipetted into another 50ml conical tube (BD, USA) and washed twice with 10ml PBS at 400xg, 22^0^C for 10 minutes followed by cell counting.

### CD4 + T cell phenotyping;

Surface staining was performed as follows: 800,000 cells of isolated CMC and PBMC were incubated at 4^0^C for 30 minutes with antibodies (with conjugated fluorochromes) targeting CD4-BV650, CD3-AF700, CCR4-PECy7, CXCR3-BV510, CCR5-PECY759, Beta7-PerCP/Cy5.5, Beta1-APC, Alpha 4-PE-Green and LIVE/DEAD-NIR-APC-Cy7 CD4. Cells were then washed in 2 ml of PBS (with 2% FBS) at 640g for 4 minutes at room temperature. Cells were fixed by adding 200μl of 1X CellFIX solution (BD, USA) and incubated for 15 minutes at 4 ^0^C wrapped in aluminum foil and washed as before. The stained fixed cell pellet was re-suspended in 200μl of PBS (with 2%FBS) and kept in the fridge (4^0^C) wrapped and acquired on an LSR II Flow Cytometer. CD3 + CD4 + T cell phenotype was identified from the isolated CMCs and PBMCs based on their size and granularity using a FSC/SSC plot (Supplementary Fig. 1A (i)) and B (i)), doublets by a FSC-A/FSC-H plot (Supplementary Fig. 1A(ii) and B (ii)), live and dead cell were separated using a Live-Dead NIR/SSCA plot (Supplementary Fig. 1A (iii) and B (iii)), and CD3:CD4 double positives identified by a CD4/CD3 plot (Supplementary Fig. 1A (iv) and B (iv)). Integrin receptor expressing CD4 + T cells were identified by gating for α4+, β1_+_ and β7 + from the CD3 + CD4 + gate using a α4+, β1 + or β7+ /CD4 + plot (Supplementary Fig. 3).

### Green Fluorescent Protein (GFP)-tagged pseudo-virus production;

GFP-tagged pseudo-virus was obtained from the “Elite study” where production was performed using, with adaptation, a method described by Nasri et al, 2014. In summary, the single cycle GFP-tagged pseudo-virus were produced by transfection of 100mg of each plasmid (envelope and backbone + transfer vector and packaging vector) as follows; CIY + VSVG (Pan tropic); CIY + ADA (R5 tropic HIV) and CIY + YU-2(R5 tropic HIV) in cultures of 100,000 cell lines per well followed by incubation for 3 days. Pseudo-virus in the cell culture supernatants were harvested and titrated by transduction of HEK 293T cells. The cells were then transduced by serial dilution of vector and the GFP expressing cells were analyzed using flow cytometry. Titrated pseudo-viruses were aliquoted in 1.0mLs and frozen until used in the infectivity assays.

### HIV-1 entry assay;

To measure susceptibility to HIV entry, 100ul of CMCs and PBMCs were spinoculated (1700xg, 2hrs, 17°C, with brakes) and cultured with either 0.5 MOIs of GFP-tagged HIV-1 pseudo-virus particles (infection well) or RPMI (mock well) in 48 flat bottomed well plates (Fisher Scientific, USA) at 37°C and 0.5% CO_2_ for 36 hours to allow viral entry to occur. Contents of each culture well were transferred into a labelled FACS tube (BD Bioscience, UK) and washed in 2mls of PBS with 2% FBS (640xg for 4 min). Cells were then surface stained with CD4-BV650, CD3-AF700, CCR5-PE-CF594, Alpha4-PE-green, Beta7-PerCP-Cy5.5, Beta 1 (CD49d)-647-APC and LD-NIR-APC-Cy7 surface marker antibodies as described above. Both Immunophenotyping and HIV entry were then measured by acquiring cells on an LSR II flow cytometer (BD Biosciences, UK). HIV viral entry was measured by the frequency of CD3 + CD4 + T cell expressing GFP. The GFP + CD3 + CD4 + T cells were gated for using a GFP+/CD4 + plot from the CD3 + CD4 + gate (Supplementary Fig. 2).

### Data analysis;

Flow cytometry data was acquired on the LSRII Flow cytometer. Data from FACSDIVA was analyzed using FlowJo software v10.11 and exported to Microsoft Excel. Statistical analysis was performed using GraphPad Prism V6 software. Comparison of CD4 + T cell phenotype frequencies and Viral entry of CD4 + T cells between the alcohol consumers and non-consumer control group was assessed using non-parametric Mann-Whitney U-test and a *P* value of less than 0.05 was considered significant at a confidence interval of 95%.

## Figures and Tables

**Table 1:** Socio-demographic characteristics of study female non-pregnant adult participants aged 18–49 recruited from fishing communities of Kigungu and Kasenyi fish landing sites of Lake Victoria, Entebbe peninsular, grouped in to the Alcohol Consumer (ALC) and the Non-alcohol consumer (NLC) groups based on the WHO-AUDIT test score of 8/40.

Participants’ Characteristic	Alcohol Consumer Group (ALC)		Non-alcohol Consumer Group (NLC)		Statistics (P Values)
**Age (Mean)**	29.93		29.88		P=0.702
**Level of Education**					
No formal education	2		1		
Primary education	11		16		
Ordinary level secondary	8		6		
Advanced secondary	3		1		P=0.825
Tertiary education	1		1		
University	1		1		
**Marital status**					
Married	11		11		P=0.497
Cohabiting	5		7		
Widowed	2		0		
Single	8		7	
**History of having had vaginal sex**	**No**	**Yes**	**No**	**Yes**	P=0.304
	0	26	1	25	
**Genital tract infection symptoms**	**No**	**Yes**	**No**	**Yes**	
Grey-Greenish vaginal discharge	21	5	5	21	P=0.947
Vaginal itching	23	3	21	5	P=0.409
Foul “fishy” vaginal odor	20	6	18	8	P=0.480
Burning urination	25	1	49	3	P=0.280
Genital sores/blisters	24	2	23	3	P=0.607

## Data Availability

Most of the datasets used and/or analyzed during the current study are included in this published article and in supplementary data files. Additional raw/primary data such as Microsoft Excel exports from Flow Jo are available from the corresponding author on request.

## List Of Abbreviations

AIDS: acquired immunodeficiency syndrome
AUDs: Alcohol Use Disorders
AUDIT: Alcohol Use Disorder Identification Test
CCR5: Cysteine-Cysteine Chemokine Receptor 5
CD: Cluster of differentiation
CMCs: Cervical Mononuclear Cells
DCs: Dendritic cells
FBS: Fetal Bovine Serum
FSC: Forward Scatter
GFP: Green Fluorescent Protein
HIV: Human Immunodeficiency Virus
IUD: Intra-Uterine Device
MARPs: Most -at – Risk Populations
PBMCs: Peripheral Mononuclear Cells
RDT: Rapid Diagnostic Test
SBS REC: Makerere University School of Biomedical Sciences Research Ethics Committee
SSC: Side Scatter
VSVG: Vesicular Stomatitis Virus Glycoprotein
WHO: World Health Organization
